# Dosimetric Impact of Intrafraction Prostate Motion and Interfraction Anatomical Changes in Dose-Escalated Linac-Based SBRT

**DOI:** 10.3390/cancers15041153

**Published:** 2023-02-10

**Authors:** Valeria Faccenda, Denis Panizza, Martina Camilla Daniotti, Roberto Pellegrini, Sara Trivellato, Paolo Caricato, Raffaella Lucchini, Elena De Ponti, Stefano Arcangeli

**Affiliations:** 1 Medical Physics Department, Fondazione IRCCS San Gerardo dei Tintori, 20900 Monza, Italy; 2 School of Medicine and Surgery, University of Milan Bicocca, 20126 Milan, Italy; 3 Department of Physics, University of Milan, 20133 Milan, Italy; 4 Elekta AB, 113 57 Stockholm, Sweden; 5 Radiation Oncology Department, Fondazione IRCCS San Gerardo dei Tintori, 20900 Monza, Italy

**Keywords:** prostate cancer, stereotactic body radiotherapy, intrafraction motion management, daily anatomy, delivered dose assessment

## Abstract

**Simple Summary:**

With an ever-growing acceptance by the radiation oncology community, stereotactic body radiation therapy (SBRT) has become an increasingly common option for localized prostate cancer in recent years. However, such high doses per fraction require the specific management of the inter- and intrafraction movements of the target. In this work, synchronized motion-inclusive dose distributions using intrafraction motion data provided by a novel electromagnetic transmitter-based device were reconstructed and recomputed on deformed CTs reflecting the CBCT daily anatomy to represent the actual delivered dose. To our knowledge, there have been no previously published studies where the dosimetric impact on the target and organs at risk (OARs) of both intrafraction prostate motion and interfraction anatomical changes was investigated together in dose-escalated linac-based SBRT. Moreover, treatments that would have been delivered without any organ motion management (non-gated) were simulated to also evaluate the dosimetric benefit of employing continuous monitoring, beam gating, and motion correction strategies.

**Abstract:**

The dosimetric impact of intrafraction prostate motion and interfraction anatomical changes and the effect of beam gating and motion correction were investigated in dose-escalated linac-based SBRT. Fifty-six gated fractions were delivered using a novel electromagnetic tracking device with a 2 mm threshold. Real-time prostate motion data were incorporated into the patient’s original plan with an isocenter shift method. Delivered dose distributions were obtained by recalculating these motion-encoded plans on deformed CTs reflecting the patient’s CBCT daily anatomy. Non-gated treatments were simulated using the prostate motion data assuming that no treatment interruptions have occurred. The mean relative dose differences between delivered and planned treatments were −3.0% [−18.5–2.8] for CTV D99% and −2.6% [−17.8–1.0] for PTV D95%. The median cumulative CTV coverage with 93% of the prescribed dose was satisfactory. Urethra sparing was slightly degraded, with the maximum dose increased by only 1.0% on average, and a mean reduction in the rectum and bladder doses was seen in almost all dose metrics. Intrafraction prostate motion marginally contributed in gated treatments, while in non-gated treatments, further deteriorations in the minimum target coverage and bladder dose metrics would have occurred on average. The implemented motion management strategy and the strict patient preparation regimen, along with other treatment optimization strategies, ensured no significant degradations of dose metrics in delivered treatments.

## 1. Introduction

Due to the unusual radiobiology of prostate cancer, with a low α/β ratio estimated to be ~1.5 Gy [1,2,3], ultra-hypofractionated, or stereotactic body radiation therapy (SBRT), treatments have become a standard option for localized prostate cancer in recent years [4,5,6,7]. In view of the decreased margins for the planning target volume (PTV), the reduced statistical averaging of setup errors (low number of fractions), and the longer delivery fraction time, such protocols require that higher doses per fraction are delivered with much greater accuracy and precision than conventional treatments in order not to jeopardize the target coverage and the sparing of surrounding organs at risk (OARs).

Since different studies [8,9,10,11,12,13] have recognized significant and unpredictable prostate motion during delivery, devices that provide real-time prostate monitoring and can be used for beam gating and patient position correction have increasingly been implemented. Among the available technologies, a novel electromagnetic (EM) transmitter-based device without surgical intervention has been shown to be a reliable and safe option to localize and monitor the prostate and the urethra during SBRT [13,14]. To limit prostate mobility and assess anatomical reproducibility throughout the treatment, strict preparation of the patient is usually necessary as well. Nevertheless, slow drifts or sudden transient movements of the prostate, as well as daily bladder and rectum volume modifications, are expected, and both may strongly affect the target coverage and OARs sparing [15,16,17,18,19,20,21,22]. Hence, their dosimetric effects should be investigated to fully understand the feasibility and safety of such extreme treatment schedules and the benefit of online motion management strategies.

Several methods have been applied to evaluate the impact of intrafraction prostate motion on dose distributions [16,23,24,25,26,27,28,29,30]. The first used approach involved the convolution of the static 3D dose matrix with probability density functions of the target motion [24]. Disregarding time information, this method cannot account for the interplay effect between the target motion and multileaf collimator (MLC) motion during beam delivery, which is characteristic of dynamic treatments such as volumetric arc therapy (VMAT) [21]. Synchronized dose reconstruction methods that establish the temporal correspondence between the target and MLC motion by incorporating real-time target motion data into the treatment plan recomputation need to be employed to achieve accurate motion-inclusive dose distributions [23,25,26,29,31].

Dose-of-the-day evaluations have been enabled by the increased availability of daily patient images; cone-beam computed tomography (CBCT) acquired before each SBRT fraction to check the actual status of the bladder and rectum and to adjust the position of the prostate gland with respect to the planned position may serve this purpose. However, an accurate dose calculation on CBCT is challenging due to the reduced image contrast, artifacts, CT Hounsfield Units (HU) fluctuations, the dependence on acquisition parameters and patient size, and smaller field-of-view (FOV) dimensions [17,32,33,34,35]. When images were not cropped and no missing patient tissue was observed, some studies [19,36] overcame these issues by obtaining the HU-to-electron density (ED) calibration curve for the specific CBCT system’s properties. In the case of a large patient size or too small FOV, methods that use synthetic CT images obtained by deforming the planning CT into the daily CBCT frame-of-reference have instead been proposed [30,37,38]. In this way, the FOV remains the same as in the planning CT, and so does the HU, thus not requiring the configuration of a different HU-to-ED curve in the treatment planning system (TPS).

In this work, synchronized motion-inclusive dose distributions using intrafraction motion data provided by the RayPilot system with RayPilot HypoCath (Micropos Medical AB, Gothenburg, Sweden) were reconstructed and recomputed on deformed CTs reflecting the CBCT daily anatomy to represent the actual delivered dose. To our knowledge, there have been no previously published studies where the dosimetric impacts on the target and OARs of both intrafraction prostate motion and interfraction anatomical changes were investigated together in dose-escalated linac-based SBRT. Moreover, treatments that would have been delivered without any organ motion management (non-gated) were simulated to also evaluate the dosimetric benefit of employing continuous monitoring, beam gating, and motion correction strategies.

## 2. Materials and Methods

### 2.1. Patient Cohort and Treatment Protocol

Thirteen patients (56 fractions) with organ-confined prostate cancer between June 2020 and May 2021 received 40 Gy in 5 fractions (*n* = 4) or 38 Gy in 4 fractions (*n* = 9) on consecutive days. Patients lay in the supine position with their arms over their chest and straight knees, and the FeetFix (CIVCO Medical Solutions, IA, USA) was the only immobilization system used for ankle fixation. A 16-French Foley catheter was used to fill the bladder with 100 cc of saline solution before the simulation and before each fraction, and a rectal micro-enema was administered as well. The PTV was obtained by applying a 2 mm isotropic expansion of the clinical target volume (CTV), defined as the prostate gland plus seminal vesicles. Treatments were planned with the Monte Carlo algorithm (1 mm grid spacing and 1% statistical uncertainty for calculation) of the Monaco TPS (Elekta AB, Stockholm, Sweden) on a VersaHD linear accelerator (Elekta AB, Stockholm, Sweden) using the VMAT technique with two 6 MV (*n* = 6) or 10 MV (*n* = 7) flattening-filter-free (FFF) arcs, and it was ensured that at least 95% of the PTV received 95% of the prescription dose. An accurate patient setup was achieved by using initial CBCT soft tissue matching. CBCT images were acquired using the fast prostate preset of 120 kV, 850 mAs, full scan, 60 s acquisition time, and FOV medium. The EM device consisted of a wired transmitter integrated into a dedicated lumen of the RayPilot HypoCath, a Foley catheter inserted into the patient’s urethra, and it provided real-time 3D prostate motion data. The shift of the transmitter was used as a surrogate for prostate motion [13]. Treatment was interrupted whenever the transmitter exceeded a 2 mm threshold in any of the three spatial directions. If the position of the prostate did not return within tolerances, the couch position was corrected after the matching of a new CBCT. The prostate trajectories resulting from this clinically implemented strategy of beam gating and motion correction are listed below as “case A”.

### 2.2. Intrafraction Prostate Trajectories and Simulation of Non-Gated Treatments

**Figure 1:**
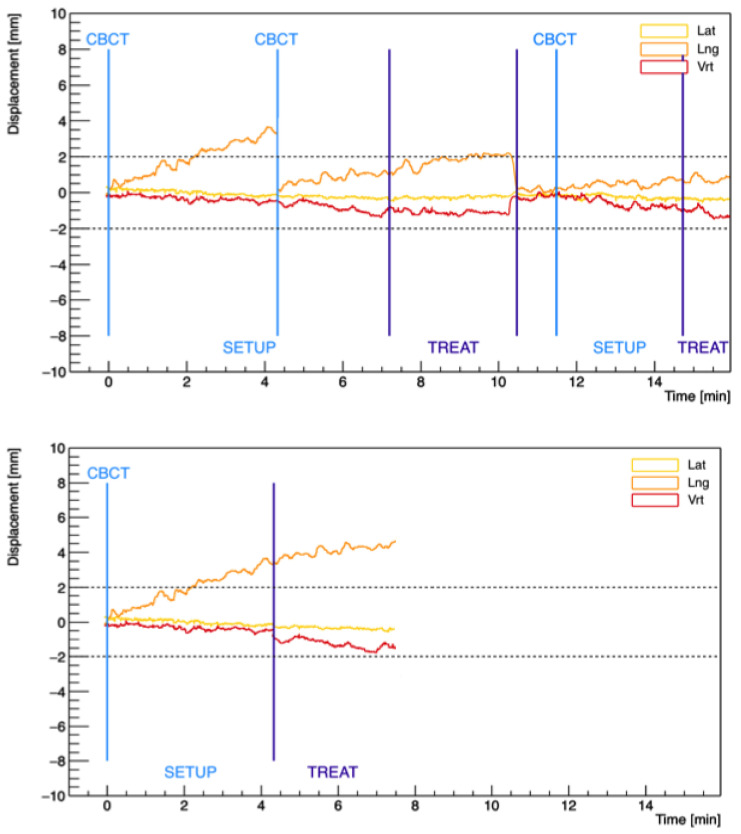
Examples of prostate trajectories in the three spatial directions obtained from case A (gated) (**top**) and case B (non-gated) (**bottom**) motion data for the same treatment fraction. The first light-blue vertical line highlights the tracking starting point (zero position) corresponding to the acquisition of the first CBCT. The following lines indicate every other acquisition of a new CBCT due to prostate displacements beyond the 2 mm threshold. The dark-blue lines separate the setup and delivery phases.

RayPilot data processing has been detailed elsewhere [13]. In short, prostate trajectories with and without beam gating and motion correction events with an update rate of 15 Hz were reconstructed and analyzed with an in-house C++ code. The trajectories that would have occurred without any organ motion management were simulated by removing all resets of the transmitter position with the acquisition of a new CBCT and by adjusting the setup and delivery duration. To this aim, a fixed duration of 3.5 min to account for image acquisition and matching on the planning CT and the real delivery time of each treatment plan were used for the setup and delivery, respectively. The prostate trajectories from this simulated scenario of non-gated treatments are referenced as “case B” hereafter. For fractions in which no interventions were required, the observed prostate trajectory data were also considered non-gated treatments. In Figure 1, examples of the prostate trajectories in case A and case B for the same treatment fraction is reported.

### 2.3. Motion-Inclusive Reconstruction Method and Dose Calculation

To evaluate the intrafraction prostate motion during SBRT delivery, synchronized motion-inclusive dose distributions were reconstructed using an isocenter shift method developed and validated by Poulsen et al. [31] and then used in other studies [21,39,40]. This method consists of dividing the patient’s original Dicom RT plan into several sub-beams formed by a certain number of control points and displacing the beam isocenter at each sub-beam according to the 3D prostate motion observed during beam delivery for each fraction. Sub-beams were created using an in-house MATLAB program (MathWorks Inc, Natick, MA, USA) to represent the part of the treatment delivery synchronized with each prostate position bin extracted from the recorded trajectories [31]. In this work, two different motion-encoded plans using data from prostate trajectories in case A and case B were reconstructed for each fraction.

To include the impact of the anatomy-of-the-day on dose distributions, synthetic CT (dCT) scans were created by deforming the planning CT on the first daily CBCT acquired during each fraction. All 56 CBCTs were exported from the X-ray Volume Imaging XVI software (Elekta AB, Stockholm, Sweden) to the Monaco TPS with the coordinate frame after registration with planning CT. Adapted contours of the main structures (i.e., CTV, urethra, rectum, bladder, and femoral heads) were checked and manually adjusted on each CBCT by an expert physician. The external structure also took into account the patient’s surface variations due to the slightly different setup positions. Deformable fusion was then performed on the CT FOV using external software (ADMIRE, research version 3.13, Elekta AB, Stockholm, Sweden), with the structures used as constraints to drive the deformation in the region of interest. In this way, inside the CBCT FOV, the resulting dCTs have the soft tissue geometry detected in the CBCT, while outside, the tissue density distributions of the planning CT were used. The accuracy of the deformation was qualitatively analyzed by evaluating the correspondence of the structure positions between dCT and CBCT and the absence of unrealistic deformations. The HU transfer between the CT and the dCT was also checked for each relevant structure. The reconstructed fraction motion-inclusive plans for case A were recalculated on the corresponding dCT using the same algorithm and calculation properties as in the original plan. The resulting dose distributions, including the contribution of both intrafraction motion and anatomical changes, were considered a good estimation of the daily dose delivered to the patients.

### 2.4. Data Analysis and Statistical Tests

Intrafraction prostate motion metrics during beam delivery were calculated in lateral, longitudinal, and vertical directions and in 3D in both case A and case B. The prostate displacements were all calculated relative to the tracking starting point position, defined at the beginning of the acquisition of the first daily CBCT.

The volumes of CTV, rectum, and bladder contoured on each daily CBCT were recorded and compared to the simulation and in between the different treatment fractions. The coefficient of variation (CV) of these structure volumes over the different treatment fractions of the same patient was also calculated.

Differences between planned and delivered doses to the target and OARs were evaluated by comparing daily fraction dose–volume histograms (DVHs) obtained from case A motion-encoded plans recomputed on each dCT with the values predicted by the original plan. The total effect of motion and anatomical changes throughout the course of treatment was also estimated by taking into account the average of the reconstructed dose parameters from all fractions of each patient. The dose-volume parameters analyzed for target structures, i.e., CTV, PTV, and the PTV shell volume around the CTV (PTV-CTV), were the mean dose, minimum dose to 99% (D99%) or 95% (D95%) of the volume and maximum dose to 2% (D2%) of the volume. For the OARs structures, the dose constraints [14] were extracted and used to assess the protocol compliance and to compare the delivered plans. This first analysis is also referenced as “comparison I” hereafter.

**Figure 2:**
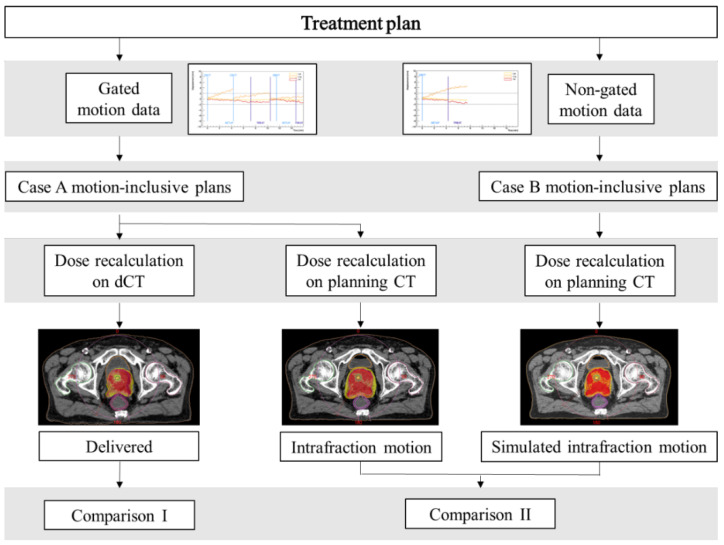
Overview of the data reconstruction process and dosimetric comparisons performed in the study.

Moreover, case A and case B motion-inclusive plans were recomputed on the planning CT images for each fraction. A direct comparison between the individual fraction and patient cumulative DVHs of the two plans was used to quantify any variation only due to the implementation (or not) of an online target motion management strategy. Thus, an estimate of which dose distributions would have been delivered to the patients if beam gating and motion correction were not employed was available. Afterward, this dosimetric analysis is referenced as “comparison II”. Figure 2 summarizes the data reconstruction process and the dosimetric comparisons performed in the study.

The Wilcoxon–Mann–Whitney signed-rank test was performed to assess the significance level, and only *p*-values less than 0.05 were considered statistically significant. The Bonferroni correction factor was also used to account for multiple testing.

## 3. Results

### 3.1. Intrafraction Prostate Motion

The prostate motion detected during beam delivery was modest in both case A and case B. Averaged over all 56 fractions, the mean [range] of the fraction mean prostate displacements were −0.2 mm [−1.5–0.8], 0.1 mm [−1.4–1.5], and −0.3 mm [−1.7–1.4] for case A and −0.3 mm [−3.1–0.8], 0.0 mm [−4.2–3.7], and −0.7 mm [−3.5–1.9] for case B in the lateral, longitudinal, and vertical directions, respectively. Positive signs indicate anterior, superior, and left displacements. The mean absolute displacements in each translational direction were 0.4 mm, 0.6 mm, and 0.7 mm in case A and 0.5 mm, 0.9 mm, and 1 mm in case B. The mean values of 3D prostate motion in the two cases were 1.1 mm [0.2–2.3] and 1.7 mm [0.2–5.1], respectively. However, the prostate would have exceeded the 2 mm margins during beam delivery in at least one spatial direction in 60% of the 25 fractions that required an intervention due to an out-of-tolerance shift. The mean prostate motion metrics over only these 25 fractions are presented in Table 1. The differences from the real shifts (case A) observed in the corresponding fractions are also reported.

### 3.2. CTV, Rectum, and Bladder Volume Changes

The median CTV volume was 47.1 cc [32.1–96.7] in the simulation and 47.8 cc [34.5–97.8] during treatment. Median volumes in the simulation were 60.9 cc [34.5–91.6] for the rectum and 131.4 cc [93.8–304.5] for the bladder, while at the time of treatment, median volumes of 61.3 cc [32.8–95.3] and 154.1 cc [86.7–335.1] were observed, respectively. Table 2 shows the mean, SD, median, and range over all patients and fractions of the percentage differences of the CTV, rectum, and bladder volumes on daily CBCTs as compared to planning CT. The average intrapatient CVs of the structure volumes with the corresponding minimum and maximum variations were also reported. None of the volume variations were statistically significant (*p* > 0.05).

**Figure 3:**
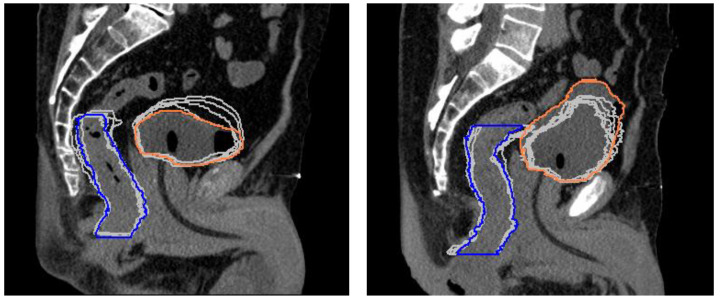
Superposition of rectum and bladder contours from the planning CT (colored lines) and each treatment fraction (gray lines) for the two patients who had the minimum (**left**) and maximum (**right**) bladder volume in the simulation.

The rectum and bladder showed larger variations both between the simulation and treatment and between the different treatment fractions of the same patient. Figure 3 shows the changes in the rectum and bladder volumes between planning and the five treatment fractions for the two patients who had the minimum and maximum bladder volumes at simulation.

### 3.3. Dosimetric Analysis

Table 3 provides the mean and range (over all fractions and patients) of the percentage differences in dosimetric parameters of delivered vs. planned dose distributions (comparison I) and between the two motion-inclusive dose distributions reconstructed using simulated case B vs. observed case A prostate motion data (comparison II). *p*-values according to the Wilcoxon signed-rank test are also reported.

#### 3.3.1. Comparison I: Delivered vs. Planned Dose Distributions

Consistent dose deficits were seen in the minimum coverage of CTV, PTV, and PTV-CTV, and the largest deviations were observed in the PTV peripheral zone; mean and maximum doses to these target structures were instead minimally affected. A median coverage of at least 93% of the prescribed dose to 99% of the CTV was achieved during treatment fractions. Considering the cumulative patient dose over the 4–5 fractions, the median CTV D99% degraded from 94% [86–96%] of the prescribed dose at planning to 93% [77–95%] at treatment. The rate of target coverage violations (D95% < 95%), along with the number of patients who did not meet the constraints in planning and treatment, is reported in Table 4. Urethra planning organ at risk volume (PRV) sparing was slightly degraded, with >1% increases in D0.035 cc and D10% observed in 48% and 36% of the fractions, respectively. In eight and three patients, at least one dose metric at the end of the treatment exceeded the planned dose to the rectum and bladder, respectively. However, only a deterioration in the protocol constraint violation rate for rectum wall D0.035 cc and two major deviations in rectum mucosa D0.035 cc were noticed at treatment completion (Table 4).

#### 3.3.2. Comparison II: Case B vs. Case A Motion-Inclusive Dose Distributions

In the motion-inclusive plans, the target dose deficits, as well as the degradations in OAR dose metrics, were marginal. None of the dosimetric parameters significantly differed from the planned values (Table A1). However, without an organ motion management strategy, significant dose reductions for CTV D99%, PTV D95%, and PTV-CTV D95% would have additionally occurred in 54%, 50%, and 70% of the fractions. For the cumulative treatments, the contribution of fractions in which prostate motion remained within the 2 mm gating threshold diminished the deterioration in the dose metrics (Table 3). Additional target coverage losses > 1% would have been experienced by four patients. Similarly, further overdoses > 1% to the urethra, rectum, and bladder would have been delivered to one, three, and seven patients, respectively. The target D95% > 95% criteria would not have been achieved in the same patients of comparison I (Table 4), but undetected out-of-tolerance prostate motion would have led to decreased minimum PTV coverage values, passing from 85% to 81% of the prescription dose in the worst case. Compared to the values presented in Table 4, one additional patient would have had a minor violation in the rectum wall D0.035 cc constraint (infringement rate equal to 23%).

## 4. Discussion

The actual delivered dose, including the contribution of both intrafraction motion and interfraction anatomical changes, was estimated by recalculating motion-encoded plans on synthetic CT deformed on the CBCT daily anatomy for dose-escalated linac-based prostate SBRT.

The motion-inclusive dose reconstruction method assumed that all target motion is due to the rigid motion of the whole patient. Small changes in the radiological path length due to changes in tissue density and the amount of tissue along the beam path were ignored, but they were not expected to compromise the accuracy of the target dose calculation in such a treatment modality. In this study, possible deformations of adjacent OARs occurring between each treatment fraction were considered to provide an accurate estimation of the effects in rectum and bladder doses [20,21,25,31]. Organ-filling variations occurring within the same daily fraction were not investigated. However, since the treatment duration was about 10 min on average, not taking into account these changes did not prevent a fair assessment of the dose delivered to OARs. It also has to be highlighted that the deformable image registration process to create the dCTs has several weaknesses itself, starting from the use of software in an undesirable “black-box” mode. According to AAPM TG-132 [41], other potential drawbacks due to different extensions, scan parameters, and image quality of the two studies being registered exist. Nevertheless, these related uncertainties are common to any commercial software and image registration process, and thus, the obtained dCTs, after being qualitatively validated, were deemed a reasonable representation of the daily organ morphology.

Our findings showed that the CTV largely differed from the simulation in only one patient, for whom a mean volume reduction of −12.6% was observed. This may be due to the cytoreductive effect of the androgen deprivation therapy (ADT) started by the patient almost six weeks before the treatment. Deformation and swelling in the prostate gland during extremely hypofractionated regimens have been observed [42,43], but our clinical schedule of 4 or 5 fractions delivered on consecutive days with ADT, received as per the standard of care [44] by 77% of the patients, seemed to have no relevant effect on prostate size. Gunnlauggsson et al. [42] found a 14% mean relative volume increase after three treatment fractions, while our mean increase at the same point was about 1%, although the different fractionation (6.1 Gy × 7), the use of magnetic resonance imaging (MRI) for the determination of prostate volume variations, and the use of ADT as exclusion criteria make these results not directly comparable. In keeping with previous experiences [15,17,19,22,36], large variations in rectum and bladder volumes likely occurred between the simulation and the daily treatment fractions. In the present study, the rectal micro-enema administered in the department shortly before each fraction appeared to be more effective than the procedure of filling the bladder with 100 cc of saline solution through the catheter in ensuring anatomical reproducibility. This may be related to the user-dependent bladder-filling procedure and the different positions that the catheter may take during its insertion on the first treatment day compared to the simulation.

A possible weakness of the current analysis is related to the challenges and thus possible uncertainties in CBCT contouring due to the poor image quality and the reduced contrast with respect to conventional CT images. We tried to minimize the inter-observer variability by leaving the contouring to the discretion of a single radiation oncologist experienced in the field. Pawloswki et al. [45] demonstrated that even though the prostate and the rectum were very sensitive to the uncertainties of CBCT contours, with volume variations up to 41% and 50%, respectively, in extreme cases, the effect on the calculated dose distributions was modest when image guidance was used. Hatton et al. [46], instead, by directly investigating the contouring accuracy between fan-beam and cone-beam CT scans, found no systematic differences in contour sizes and shapes.

Our dosimetric results from the delivered vs. planned dose distributions (Table 3, comparison I) showed that both intrafraction prostate motion and daily anatomical deformations minimally affected the mean and maximum doses to the target structures. The minimum doses, instead, more largely and frequently differed from the planned values. However, it must be pointed out that the results were a function of the chosen dosimetric endpoints: D99% of the CTV and D95% of the PTV were considered surrogates of the minimum target coverage in this study since they are already used as the treatment plan acceptability criteria at our institution. Moreover, since no other studies have performed dose recalculations accounting for both motion and daily anatomy in such treatments to our knowledge, these findings are difficult to compare to the existing literature. Langen et al. [25] investigated only the effect of motion during tomotherapy treatments and found an unexpected similarity between the observed D95% changes in the prostate and PTV, suggesting that the effects of motion are not necessarily restricted to the periphery of the PTV and, thus, that margins may not completely protect the target from a geographic miss. Conversely, we found that major degradations in the minimum but also the mean dose occurred in the PTV shell around the CTV, indicating the positive impact of the margins on ensuring the target coverage.

Margins were not the only implemented strategy to minimize a potential target miss: a strict patient preparation regimen, soft-tissue CBCT matching, intrafraction organ motion management, robust treatment planning, and fast FFF-beam delivery also played a crucial role. The registrations were focused on the prostate gland and were always performed to obtain the best overlap between the HypoCath and the urethra PRV delineated in the planning CT. This refinement allowed us to achieve the urethral sparing at which our plans aimed and to prevent a possible underdose to the surrounding CTV due to the lower prescribed dose to the urethra. Our findings showing no relevant differences in urethra dosimetry confirm the efficacy of this strategy.

Still, it is noteworthy that only translational errors in the target position were corrected, while rotations were not. Ma et al. [43] investigated the dosimetric impact of uncorrected interfraction rotations in the prostate and proximal seminal vesicles separately and found that the seminal vesicles exhibited inferior target dosimetry results to those of the prostate due to their considerable variations in the relative angle, both between the two lobes and relative to the vertical axis. By including the seminal vesicles in CTVs, target coverage reductions might have been at least partly affected by their rotational shifts. However, although severe target degradations as large as about 15–20% were observed in some individual fractions, they were infrequent, and their effect on the cumulative dose was smoothed with the number of fractions. The DVH analysis showed that the coverage of the prostate gland was satisfactory, with a median cumulative CTV coverage of 93% of the prescribed dose and only two patients who experienced a CTV dose deficit of more than 5%.

Due to the consistent daily variations in bladder and rectal volumes, the delivered doses to these OARs did not meet the treatment plan provisions in most fractions, as reported by many other studies [15,19,20,22]. The pattern of these differences, however, is hard to explain and to compare to the literature since the synergic effects of random volume and shape variations, organ motion, and changes in dynamic beam parameters may cause unpredictable differences in dose distributions and have never been explored. Wahl et al. [20] demonstrated that significantly higher doses during prostate SBRT were received by the rectum, while the bladder modestly differed from the original plan’s dose. Instead, for our patient cohort and treatment delivery technique, the rectum received higher-than-planned doses in less than 40% of the fractions, and the bladder doses exhibited the largest and most frequent variations.

The minimal differences resulting from the motion-inclusive plans using case A prostate trajectories compared to the planned values implied that the intrafraction prostate motion marginally contributed in the scenario of gated treatments with a 2 mm threshold (Table A1). In our treatments, even with an accurate correction strategy using continuous tracking, beam gating, and target repositioning, the dosimetric variability was still not completely compensated, as similarly reported by two other works [15,20], indicating that the major contribution to both target and OARs discrepancies came from interfraction anatomical deformations. This emphasizes the importance of assessing the effects of daily variations in non-rigid body anatomy and led us to work toward especially improving the bladder preparation procedure through more specific instructions and different filling modalities. The current study determined that only a few patients failed at least one of the rectum and bladder constraints at treatment completion (Table 4). However, the larger doses delivered to the rectum and the bladder did not necessarily correlate with increased toxicity. The early treatment outcomes for this patient group were previously published [14], showing that no Grade 2 or higher gastrointestinal (rectal) or genitourinary side effects occurred within 90 days from the end of the treatment. At a median follow-up of 18 months, only two late Grade 2 side effects were observed. Those differences slightly affected the target coverage, and indeed, biochemical control was not compromised.

The recent randomized trial MIRAGE [47] reported a significantly lower toxicity profile with MR-guided daily adaptive RT (MRgRT) vs. CT-guided SBRT in prostate cancer. The 4 mm margins and delivery without intrafraction monitoring used in the CT arm, however, make a direct comparison with the current study findings difficult. Moreover, Nicosia et al. [36], evaluating the dosimetric differences between MRgRT and image-guided SBRT with or without fiducial markers, found the highest accuracy of MRgRT as compared to SBRT without fiducials but minimal or absent differences with fiducials. Interfraction anatomical variation issues may be better overcome in an online adaptive setting, but the use of internal markers, fiducials, or transmitters, along with the other abovementioned planning and delivery techniques, keeps linac-based prostate SBRT valid and suitable. Similar results in terms of a reduction in the target coverage and an increase in the OAR constraint violation rate in a recent dosimetric analysis for 15 patients by Brennan et al. [48], using different real-time MRI scans to estimate the true delivered dose, support this conclusion.

A direct comparison between case B and case A motion-inclusive dose distributions showed that the implemented strategy of organ motion mitigation was effective at preventing larger target dose deficits and bladder overdoses for fractions that would have had higher prostate displacements. Similarly, Colvill et al. [16] demonstrated that gated dose calculations with a 3 mm–5 s threshold would have led to improvements in all CTV D99% and PTV D95% values with respect to delivered non-gated treatments. The results of the current study, however, showed that even in non-gated treatments, deteriorations in dose parameters would not have been as relevant, with only one additional patient experiencing a constraint violation for the rectum wall. This is explained by the fact that prostate motion beyond the tolerances would have been rare even in case B due to the short treatment times enabled by VMAT-FFF plans. Indeed, the likelihood of prostate motion has been demonstrated to increase with increasing treatment time. Vanhanen et al. [21] also concluded that continuous motion monitoring and gating may have a greater beneficial effect with treatment techniques associated with a longer beam delivery time. However, intratreatment position correction, along with accurate CBCT-based interfraction motion correction, ensured superior results for every fraction and patient. Hence, they are recommended to use in dose-escalated treatments with an increased risk of OARs adverse events and a further reduction in PTV margins.

## 5. Conclusions

For the first time, the dosimetric impacts of intrafraction motion and interfraction changes have been investigated together in dose-escalated linac-based prostate SBRT. The implemented organ motion management strategy and the strict patient preparation regimen, along with current PTV margins, the robustness of the original treatment plans, soft-tissue CBCT matching, and fast FFF-beam delivery, ensured there were no significant degradations of target and OARs dose metrics. Non-gated treatments would have resulted in larger target dose deficits and bladder overdoses in some fractions. Thus, continuous monitoring, beam gating, and motion correction are recommended to safely deliver such extreme hypofractionated treatments.

## Figures and Tables

**Table 1:** Mean, mean absolute “abs”, and range of intrafraction prostate displacements during beam delivery in case B averaged over the 25 fractions that required an intervention due to excessive prostate motion. Values are provided in each of the three spatial directions and in 3D and are all expressed in mm. Mean differences from real shifts observed in the corresponding fractions are indicated by “diff”.

Direction	Mean [Range]	Mean Abs	Mean Diff [Range]	Mean Abs Diff
Lateral	−0.5 [−3.1–0.8]	0.7	−0.3 [−1.6–0.3]	0.3
Longitudinal	−0.4 [−4.2–3.7]	1.4	−0.4 [−2.8–2.4]	0.7
Vertical	−1.2 [−3.5–1.9]	1.6	−0.7 [−1.8–0.7]	0.7
3D	2.6 [0.7–5.1]		1.3 [0.3–2.8]	

**Table 2:** Mean, standard deviation (SD), median, and range of percentage differences in CTV, rectum, and bladder volumes between daily fraction CBCTs and planning CTs. The values were averaged over the means of each patient for CTV and over all 56 fractions for rectum and bladder. Average intrapatient CV and range of the structure volumes are also reported.

	Mean (SD)	Median [Range]	CV [Range]
CTV	−1.3% (4.0)	−0.1% [−12.6–2.2]	2.3% [0.4–4.2]
Rectum	−3.5% (10.1)	−3.8% [−19.9–27.3]	5.7% [2.3–8.5]
Bladder	+8.9% (42.0)	+8.7% [−60.9–117.6]	19.6% [13.5–28.3]

**Table 3:** Mean and range (over all fractions and patients) of the percentage differences in target and OARs dosimetric parameters between the delivered and planned dose distributions (comparison I) and between the two motion-inclusive dose distributions reconstructed from case B and case A prostate motion data (comparison II). In the latter, for the individual fractions, only the 25 fractions that required an intervention due to prostate motion outside the tolerances were considered, while the cumulative mean differences were obtained by summing the gated and non-gated fractions as required. Statistically significant differences (*p* < 0.05) are highlighted.

		Comparison I			Comparison II		
	Metrics	Individual Fractions	*p*-Value	Cumulative Treatments	Individual Fractions	*p*-Value	Cumulative Treatments
CTV	Dmean	−0.5% [−2.0–1.2]	0.416	−0.5% [−1.4–0.2]	−0.2% [−2.3–0.8]	0.312	0.0% [−0.5–1.2]
	D99%	−3.0% [−18.5–2.8]	0.001	−3.1% [−13.2–0.5]	−2.8% [−16.3–1.1]	0.020	−1.3% [−8.3–0.2]
	D2%	−0.4% [−1.6–2.8]	0.028	−0.4% [−1.2–0.1]	−0.1% [−4.2–0.6]	1.000	+0.1% [−0.7–1.3]
						0.284	
PTV	Dmean	−0.7% [−2.9–1.2]	0.059	−0.6% [−1.9–0.2]	−0.4% [−2.1–0.8]	0.284	−0.1% [−0.6–1.4]
	D95%	−2.6% [−17.8–1.0]	0.000	−2.7% [−11.9–−0.2]	−2.4% [−11.9–0.9]	0.002	−1.0% [−5.3–0.6]
	D2%	−0.4% [−1.5–2.7]	0.074	−0.3% [−1.1–0.2]	−0.4% [−4.1–0.6]	0.926	+0.1% [−0.8–1.4]
PTV—CTV	Dmean	−1.2% [−5.8–1.3]	0.059	−1.2% [−3.9–0.5]	−0.8% [−3.6–0.6]	0.270	−0.2% [−1.5–1.8]
	D95%	−4.8% [−27.3–6.4]	0.001	−4.9% [−19.3–3.6]	−5.6% [−23.7–1.9]	0.013	−2.0% [−8.9–1.1]
Urethra PRV	D0.035 cc	+1.0% [−1.6–5.6]	0.046	+1.1% [−0.7–2.2]	+0.4% [−5.3–2.9]	0.333	+0.2% [−0.6–0.8]
	D10%	+0.7% [−1.2–4.9]	0.158	+0.7% [−0.6–1.3]	+0.6% [−5.0–6.3]	0.312	+0.2% [−0.7–1.0]
Rectum	D5%	−4.7% [−35.9–24.6]	0.163	−4.7% [−27.7–12.0]	−4.3% [−30.8–13.0]	0.240	−1.8% [−9.6–5.7]
	D10%	−5.0% [−41.7–31.7]	0.371	−5.1% [−33.5–14.6]	−4.6% [−33.8–27.5]	0.248	−2.0% [−10.5–6.6]
	D20%	−3.6% [−38.4–39.0]	0.514	−3.7% [−31.3–15.5]	−4.8% [−31.2–38.0]	0.343	−2.0% [−11.5–7.3]
	D50%	−1.5% [−24.1–38.8]	0.792	−1.5% [−18.9–16.1]	−1.9% [−22.1–25.1]	0.742	−0.9% [−8.8–8.2]
Rectum wall	D0.035 cc	−0.8% [−13.5–12.6]	0.921	−0.5% [−5.9–8.7]	−3.0% [−25.2–4.7]	0.177	−1.4% [−12.0–3.3]
Rectum mucosa	D0.035 cc	+0.8% [−27.1–33.9]	0.560	+1.0% [−10.5–22.6]	−3.9% [−30.3–8.6]	0.338	−1.8% [−10.9–4.7]
Bladder	D0.035 cc	−0.8% [−7.0–1.2]	0.123	−0.8% [−2.4–0.4]	+0.3% [−6.3–3.0]	0.445	+0.3% [−1.2–3.2]
	D10%	−4.6% [−44.6–38.0]	0.077	−4.6% [−27.6–20.5]	+3.1% [−13.8–24.6]	0.421	+0.9% [−4.2–5.4]
	D40%	+2.6% [−74.0–319.9]	0.019	−0.9% [−52.7–234.4]	+11.6% [−22.8–83.5]	0.680	+3.2% [−9.8–17.7]

**Table 4:** Protocol dose constraint infringement rate for target and OARs, along with the number of patients who failed in planning and treatment, for the delivered treatments.

		Planning			Treatment	
Dose Constraints	Patients Failing	Infringement Rate	Major Deviations	Patients Failing	Infringement Rate	Major Deviations
PTV D95% < 95%	0	0%	-	7	54%	1
CTV D95% < 95%	-	-	-	4	31%	-
Rectum wall D0.035 cc	0	0%	-	2	15%	-
Rectum mucosa D0.035 cc	6	46%	-	5	38%	2
Bladder D40%	1	8%	1	1	8%	1

## Data Availability

Research data are stored in an institutional repository and can be shared upon reasonable request to the corresponding author.

## Appendix A

**Table A1:** Mean and range (over all fractions and patients) of the percentage differences in target and OARs dosimetric parameters between intrafraction motion-inclusive recalculations considering the observed case A prostate motion data and the planned dose distributions. *p*-values (*p* < 0.05) for the individual fractions’ dose parameters are also reported.

	Metric	Individual Fractions	*p*-Value	Cumulative Treatments
CTV	Dmean	+0.1% [−0.7–2.3]	0.522	+0.1% [−0.1–0.5]
	D99%	−0.2% [−5.1–5.1]	0.392	+0.0% [−1.8–1.1]
	D2%	+0.3% [−0.4–4.4]	0.086	−0.4% [−0.7–0.1]
PTV	Dmean	0.0% [−0.7–2.0]	0.875	0.0% [−0.4–0.4]
	D95%	−0.4% [−3.7–1.4]	0.136	−0.2% [−1.6–0.8]
	D2%	+0.4% [−0.4–4.3]	0.056	−0.4% [−0.7–0.1]
PTV—CTV	Dmean	−0.1% [−1.5–1.0]	0.907	−0.1% [−1.0–0.3]
	D95%	−1.0% [−12.1–7.1]	0.337	−1.1% [−7.8–2.5]
Urethra PRV	D0.035 cc	+0.7% [−0.8–6.4]	0.168	−0.2% [−0.6–0.4]
	D10%	+0.6% [−0.9–5.4]	0.175	+0.1% [−0.3–0.9]
Rectum	D5%	−1.7% [−14.4–11.5]	0.419	−2.5% [−8.8–3.1]
	D10%	−2.3% [−16.7–14.2]	0.526	−2.9% [−9.7–3.8]
	D20%	−2.9% [−39.8–13.6]	0.592	−3.1% [−10.4–4.9]
	D50%	−1.3% [−12.5–6.2]	0.852	−1.8% [−7.6–2.2]
Rectum wall	D0.035 cc	−0.6% [−7.9–5.3]	0.584	−1.6% [−4.8–1.7]
Rectum mucosa	D0.035 cc	−1.1% [−13.5–14.0]	0.507	−2.3% [−8.3–3.3]
Bladder	D0.035 cc	+0.3% [−1.9–3.3]	0.580	−0.2% [−1.1–1.1]
	D10%	+0.2% [−11.7–11.4]	0.926	+1.2% [−4.9–7.5]
	D40%	+1.4 [−18.7–37.7]	0.907	+5.1% [−7.8–25.1]

Only in 25% of all fractions did the PTV coverage drop below 95% of the prescribed dose, with a minimum coverage value of 92%. The percentage of fractions with more than 90% minimum CTV coverage was 95%. Degradations larger than 1% in CTV D99% and PTV D95% were seen only for three patients. The cumulative effect of intrafraction prostate motion would have caused violations of the minimum PTV coverage requirement (D95% > 95%) in only one patient, whose target would have been covered by 94% of the prescribed dose at minimum. Overdoses >5% to the urethra, rectum, and bladder were observed in some treatment fractions. In the motion-inclusive cumulative plans, differences no larger than 1% with respect to planned values would have been observed for the urethra. Only three patients would have had rectum dose increases >1% in at least one parameter at the end of treatment, while for the bladder, the same occurred in nine patients. With a 2 mm threshold beam gating strategy implemented, no differences in the OARs protocol dose constraint violation rate would have been observed due to the intrafraction motion of the prostate.
